# Immediate and delayed placement of the intrauterine device after abortion: a systematic review and meta-analysis

**DOI:** 10.1038/s41598-024-62327-1

**Published:** 2024-05-18

**Authors:** Ying Lou, Shanshan Tang, Zhumei Sheng, Hongqin Lian, Jingjing Yang, Xuejing Jin

**Affiliations:** 1 Women Healthcare Department, CiXi Maternity and Child Health Care Hospital, Cixi, 315300 Zhejiang China; 2 Gynecology Department, Hangzhou Women’s Hospital, Hangzhou, 310000 Zhejiang China; 3 Women Healthcare Department, Hangzhou Women’s Hospital, Hangzhou, 310000 Zhejiang China; 4 Gynecology Department, CiXi Maternity and Child Health Care Hospital, Cixi, 315300 Zhejiang China; 5 Medical Department, CiXi Maternity and Child Health Care Hospital, Cixi, 315300 Zhejiang China; 6 Reproductive Endocrinology Center, Hangzhou Women’s Hospital, 369 Kunpeng Road, Shangcheng District, Hangzhou, 310000 Zhejiang China

**Keywords:** Intrauterine device (IUD), Immediate insertion, Delayed insertion, Abortion, Meta-analysis, Health care, Medical research

## Abstract

This article aims to report the comprehensive and up-to-date analysis and evidence of the insertion rate, expulsion rate, removal rate, and utilization rate of immediate placement of intrauterine devices (IUDs) versus delayed placement after artificial abortion. PubMed, Embase, Cochrane, Web of Science, CNKI, and Wanfang databases were comprehensively searched up to January 12, 2024 for studies that compared immediate versus delayed insertion of IUDs after abortion. The evaluation metrics included the number of IUD insertion after surgical or medical abortions, the frequency of expulsion and removal at 6 months or 1 year, the number of continued usage, pain intensity scores, the number of infections, the duration of bleeding, and instances of uterine perforation during or after IUD insertion. Ten randomized controlled articles were eligible, comprising 11 research projects, of which 3 projects involved the placement of an IUD after surgical abortion, and 8 projects involved the placement of an IUD after medical abortion. This included 2025 patients (977 in the immediate insertion group and 1,048 in the delayed insertion group). We summarized all the extracted evidence. The meta-analysis results indicated that for post-surgical abortions, the immediate insertion group exhibited a higher IUD placement rate than the delayed insertion group. After medical abortions, the immediate insertion group showed higher rates of IUD placement, utilization, and expulsion at 6 months or 1 year. The two groups showed no statistically significant differences in the removal rate, post-insertion infection rate, pain scores during insertion, and days of bleeding during the follow-up period. Compared to delayed placement, immediate insertion of IUDs can not only increase the usage rate at 6 months or 1 year but also enhance the placement rate.

## Introduction

Abortion emerges as a prominent concern in the realm of global health, with varying incidence rates and backgrounds worldwide^1^. It is of utmost importance to address the consequences of abortion, as it significantly increases morbidity and mortality in pregnant and postpartum women^2^. Effective contraception is important in preventing unintended pregnancies and subsequent abortions^3^. In this regard, intrauterine devices (IUDs) are becoming more popular due to their effectiveness and long-term protection^4^, indicating a trend towards more dependable contraceptive options.

Despite the acknowledged advantages of IUDs, the placement process has consistently posed challenges that require continuous attention^5^. Traditional IUDs are placed based on the menstrual cycle, which may lead to postponement in the initiation of effective contraception. Even though these delays seem insignificant, they present a potential threat of unintended pregnancies, especially in populations with limited access to medical services^6^. In addition, the appropriate time of IUD placement is crucial to the effectiveness and patient comfort, and inappropriate timing possibly increases the risks of expulsion and discomfort^7^.

The appropriate timing of IUD placement after an abortion is a significant and evolving topic of discussion^8,9^. The primary focus is to compare the successful rates of placement, expulsion, removal, and continued usage between immediate IUD insertion and delayed insertion after abortions during follow-up. Additionally, the results of IUD insertion are significantly impacted by different abortion manners, including surgical and medical abortions, and the gestational age at abortion, whether in early or mid-pregnancy^10^. These factors are crucial for assessing the practicality and acceptability of IUDs for post-abortion contraception.

This systematic review and meta-analysis intended to investigate the outcomes of immediate versus delayed IUD placement following abortion, with a specific focus on the effect of the abortion methods and gestational age at abortion. We hope to provide clear evidence-based insights to guide clinical practices and decision-making in reproductive health, thereby optimizing the use of IUDs in post-abortion contraceptive services.

## Materials and methods

### Literature search

This systematic study was prospectively registered in PROSPERO (CRD42023425587) and reported according to the Preferred Reporting Items for Systematic Reviews and Meta-Analyses (PRISMA) 2020 statement^11^. Online Resource S1 shows the PRISMA 2020 checklist.

PubMed, Embase, Cochrane, Web of Science, CNKI, and the Wanfang databases were systematically searched up to January 12, 2024, for literature that compared the successful insertion rate, expulsion rate, removal rate, and usage rate of immediate versus delayed IUD placement after abortion. Given the different outcomes of different abortion methods, we classified the abortion methods into surgical abortion and medical abortion and analyzed their outcomes. We retrieved articles published after 2003 for our up-to-date analysis. Similar meta-analyses^12^ were conducted before, so we retrieved articles from the past 20 years. The detailed search strategy is presented in Online Resource S2. Additionally, we manually reviewed the reference lists of all qualified studies. Two reviewers independently conducted the search and evaluation of included studies, and any discrepancies in the literature search were resolved through discussion to reach a consensus.

## Inclusion and exclusion criteria

The following criteria were used to include studies: (1) study design: randomized controlled trials (RCTs), (2) study population: adult females who underwent surgical or medical abortion and received IUD placement for contraception, (3) comparative studies of immediate (< 10 days after abortion) versus delayed insertion of IUDs (> 2 weeks after abortion), (4) surgical abortion is to extract the gestational sac through uterine aspiration, while medical abortion is to completely discharge the gestational sac using drugs (such as Mifepristone or Misoprostol); the gestational age at abortion involves early pregnancy abortion (gestational age 64–84 days) and mid-pregnancy abortion (gestational age 85–196 days)^13^, (5) at least one outcome measure was assessed, such as the successful insertion rate, expulsion rate, removal rate, pain scores during insertion, infection rate after IUD insertion, duration of post-insertion bleeding, retention rate at 6 months or 1 year, and other complications, and (6) with available and sufficient data of weighted mean difference (WMD), standardized mean difference (SMD), or to calculate relative risk (RR).

Duplicates, comments, case reports, reviews, meta-analyses, editorials, unpublished manuscripts, letters, conference abstracts, and articles not in English or Chinese were excluded.

### Data extraction

Two reviewers independently extracted the data. Any disagreements were resolved by a third reviewer who made the final decision. The data extracted from the included studies were as follows: publication year, first author, study duration, country, registration number, sample size, study design, age, body mass index (BMI), gestational age at medical abortion (in days), past pregnancy(≥ 1), parity(≥ 1), prior abortion(≥ 1), number of successful IUD insertion (the IUD was inserted after the expulsion of the conception product in both the immediate and delayed groups), number of IUD expulsions and removals at 6 months or 1 year, number of IUD users at 6-months or 1-year follow-up, number of infections after insertion, pain scores during insertion, duration of post-insertion bleeding, and number of cases with uterine perforation. When continuous variables were reported as medians or interquartile ranges, a calculator that includes sample size was utilized to harmonize the varying data representations in the literature and the data were ultimately expressed as mean and standard deviation^14,15^. In cases of missing or unreported data in the studies, we attempted to contact the respective authors to acquire complete data, if available.

### Quality assessment

The quality assessment of the eligible RCTs was performed in accordance with the Cochrane Handbook for Systematic Reviews of Interventions 5.1.0. The evaluation involved seven domains, including the blinding of participants and staff, the creation of random sequences, the blinding of outcome assessment, the concealment of allocations, the use of selective reporting, the use of incomplete outcome data, and additional sources of bias^16^. Each facet of the study was assessed for bias, classified as low, high, or unclear risk. Studies with a greater number of domains deemed “low risk” were regarded as higher quality. Two reviewers independently assessed the quality and level of evidence of the included studies, and any discrepancies were resolved through discussion.

### Statistical analysis

Evidence synthesis was conducted using Review Manager 5.4 (Cochrane Collaboration, Oxford, UK). Furthermore, WMD and SMD were used as effect sizes for continuous variables, and RR for dichotomous variables. All effect sizes were reported with 95% confidence intervals (CIs). Moreover, the heterogeneity of the included studies was measured using the chi-square (C^2^) test and quantified by the I-squared (I^2^) statistic^17^. A C^2^ p-value < 0.05 or I^2^ > 50% was defined as significant heterogeneity. Due to heterogeneity across the studies, a random-effects model was adopted to combine WMD, SMD, or RR.

**Figure 1:**
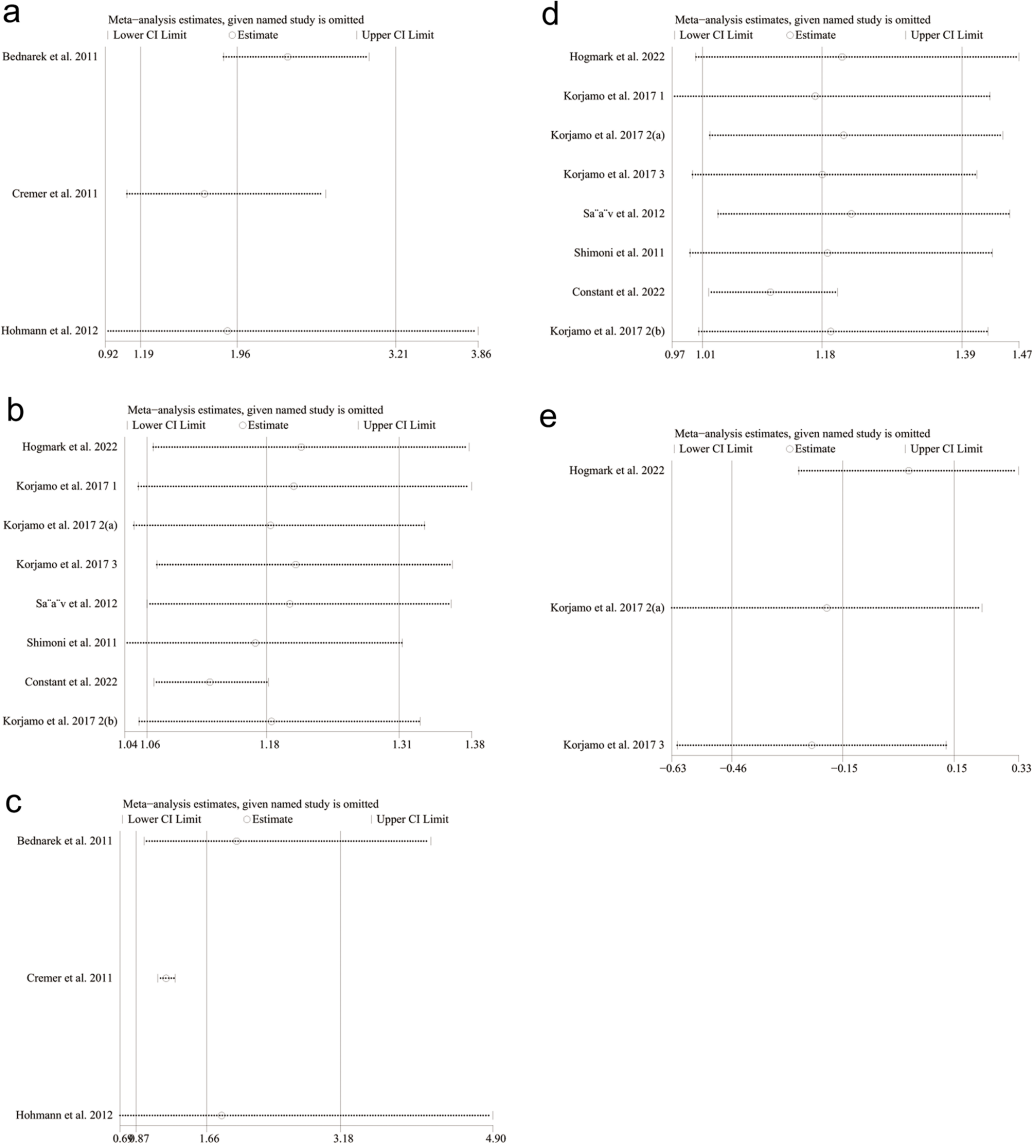
Sensitivity analysis of (**a**) IUD inserted (after surgical abortion). (**b**) IUD inserted (after medical abortion). (**c**) IUD used at 6 months (after surgical abortion). (**d**) IUD used at 6 months or 1 year (after medical abortion). (**e**) Pain score at IUD insertion (VAS or NRS) after medical abortion.

In view of the influence of abortion methods on the outcomes, we subgrouped the methods into surgical abortion and medical abortion for separate analyses. Based on different abortion methods, subgroup analyses were conducted according to gestational age, including early pregnancy abortion (gestational age 64–84 days) and mid-pregnancy abortion (gestational age 85–196 days). Subgroup analyses based on IUD type and region after medical abortion were also performed. The results are shown in Table 1. One-way sensitivity analyses were also conducted to assess the impact of the included studies on the overall outcomes, particularly in cases of significant heterogeneity, as depicted in Fig. 1. Funnel plots were created using Review Manager 5.4 to visually evaluate publication bias. Additionally, Egger's regression test^18^ was implemented by using Stata 15.0 (Stata Corp, College Station, TX, USA) for outcomes reported in three or more included studies. For publication bias, a p-value of 0.05 or lower was interpreted as statistically significant.

**Table 1:** Subgroup analysis of intrauterine device use and adverse reactions after medical abortion.

Subgroup	IUD inserted				IUD expelled at 6 months or 1 year				IUD removed at 6 months or 1 year			
	Study	RR [95% CI]	*P* value	*I*^2^	Study	RR [95% CI]	*P* value	*I*^2^	Study	RR [95% CI]	*P* value	*I*^2^
Total	8	1.18 [1.06, 1.31]	0.002	82%	6	2.08 [1.42, 3.05]	0.0002	0%	6	1.10 [0.52, 2.31]	0.81	35%
Type of IUD												
LNG-IUS	4	1.22 [1.06, 1.19]	< 0.0001	0%	4	2.42 [1.56, 3.77]	< 0.0001	0%	4	0.64 [0.32, 1.27]	0.20	0%
Cu-IUD	2	2.19 [0.49, 9.67]	0.30	97%	1	1.37 [0.52, 3.59]	0.52		1	2.39 [0.86, 6.68]	0.10	
LNG-IUS or Cu-IUD	2	1.07 [1.00, 1.15]	0.05	0%	1	1.31 [0.39, 4.39]	0.67		1	1.74 [0.46, 6.63]	0.42	
Region												
Africa	1	3.89 [2.32, 6.52]	< 0.00001									
Europe	6	1.10 [1.05, 1.15]	< 0.0001	0%	5	2.25 [1.49, 3.41]	0.0001	0%	5	0.81 [0.41, 1.59]	0.53	7%
America	1	1.27 [1.12, 1.44]	0.0002		1	1.37 [0.52, 3.59]	0.52		1	2.39 [0.86, 6.68]	0.10	

Subgroup	IUD used at 6 months or 1 year				Infection				Pain score at IUD insertion (VAS or NRS)			
	Study	RR [95% CI]	*P* value	*I*^2^	Study	RR [95% CI]	*P* value	*I*^2^	Study	SMD [95% CI]	*P* value	*I*^2^
Total	8	1.18 [1.01, 1.39]	0.04	74%	7	1.30 [0.66, 2.56]	0.45	0%	3	− 0.15 [− 0.46, 0.15]	0.33	52%
Type of IUD												
LNG-IUS	4	1.19 [1.06, 1.33]	0.002	0%	3	1.39 [0.70, 2.79]	0.35	0%	2	0.03 [− 0.27, 0.33]	0.86	0%
Cu-IUD	2	1.63 [0.80, 3.29]	0.18	92%	2							
LNG-IUS or Cu-IUD	2	1.03 [0.91, 1.15]	0.66	0%	2	0.29 [0.01, 7.00]	0.45		1	− 0.39 [− 0.65, − 0.12]	0.004	
Region												
Africa	1	1.55 [0.67, 3.60]	0.30	96%								
Europe	6	0.10 [0.99, 1.22]	0.07	26%								
America	1	1.15 [0.91, 1.45]	0.24									

Subgroup	Number of bleeding or spotting days during the reference period											
	Study	SMD [95% CI]	*P* value	*I*^2^								
Total	4	0.07[− 0.14,0.28]	0.53	0%								
Type of IUD												
LNG-IUS	2	0.12 [− 0.22, 0.45]	0.49	0%								
Cu-IUD	1	0.21 [− 0.17, 0.60]	0.28									
LNG-IUS or Cu-IUD	1	− 0.12 [− 0.49, 0.24]	0.51									
Region												
Africa												
Europe	3	0.01 [− 0.24, 0.26]	0.95	0%								
America	1	0.21 [− 0.17, 0.60]	0.28									

*IUD* intrauterine device, *LNG-IUS* levonorgestrel intrauterine system, *Cu-IUD* copper intrauterine device, *RR* relative risk, *SMD* standardized mean difference, *CI* confidence interval.

### Ethics approval

In accordance with local legislation and institutional requirements, this study did not require ethical review and approval of human subjects.

## Results

### Literature search and study characteristics

**Figure 2:**
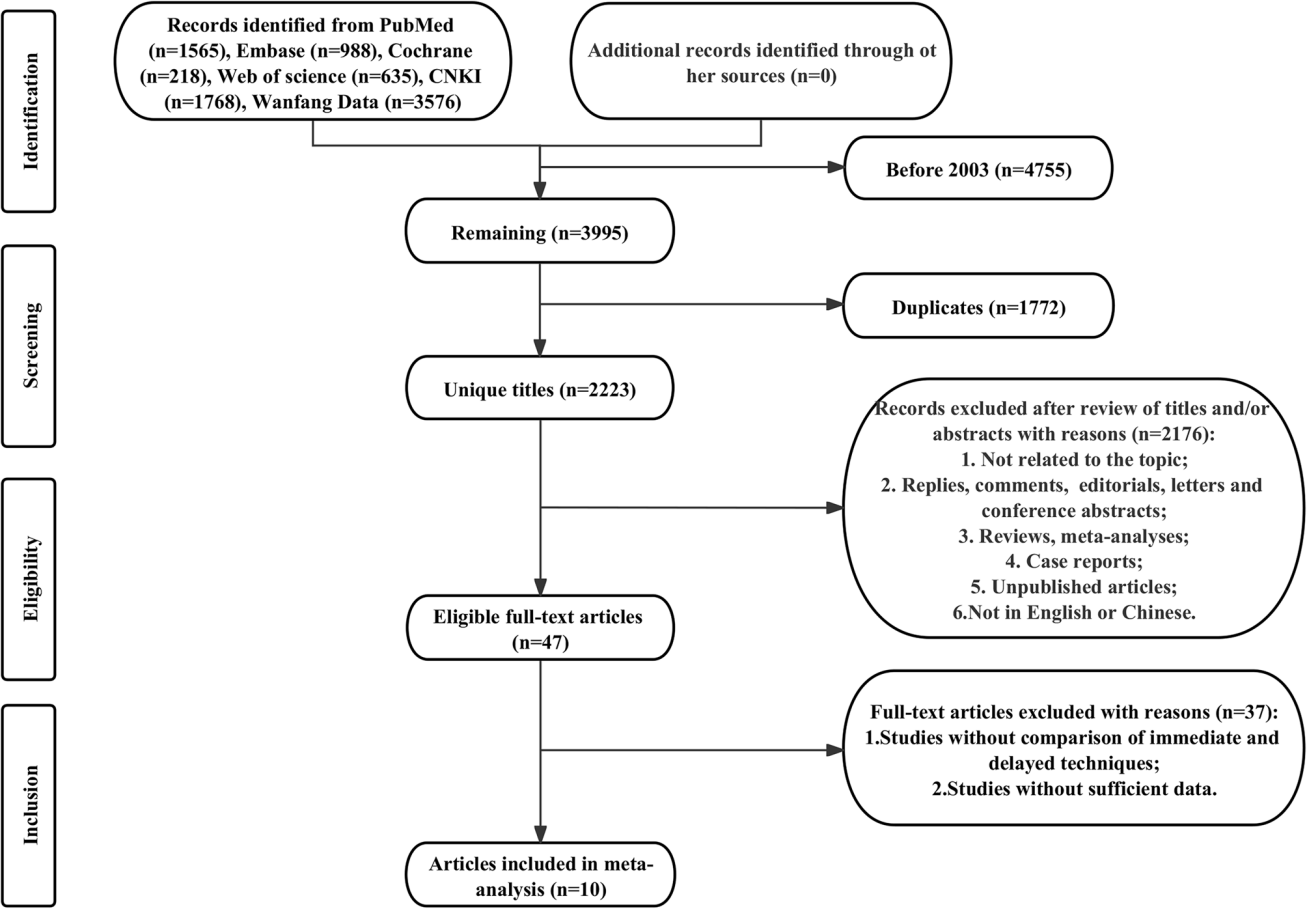
Flowchart of the systematic search and selection process.

The process of systematic search and selection is exhibited in Fig. 2. A total of 8750 articles were identified through systematic literature searches in PubMed (n = 1565), Embase (n = 988), Cochrane (n = 218), Web of Science (n = 635), CNKI (n = 1768), and Wanfang database (n = 3576). After removing duplicates and papers published before 2003, the abstracts and titles of the remaining 2223 papers were reviewed. Finally, 10 full-text articles were included in the meta-analysis, involving 2025 patients (977 in the immediate placement group and 1,048 in the delayed placement group)^8,9,19–26^. All 10 articles were RCTs.

**Figure 3:**
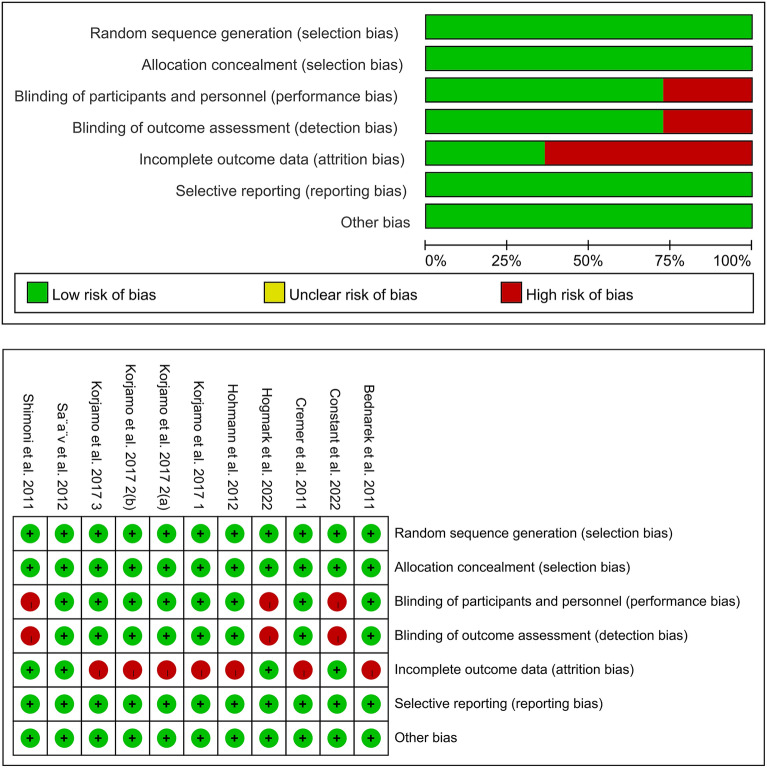
Risk of bias graph and summary.

As the study by Korjamo et al.^24^ assigned women requesting IUD placement after abortion into two groups based on gestational age (64–84 days and 85–140 days), we regarded it as two separate studies for analysis. Therefore, this meta-analysis included 11 RCTs. The quality assessment results of all eligible studies are provided in Fig. 3. The study characteristics, including the study period, location, sample size, specific timing of IUD placement, type of IUDs, and maximum follow-up duration are displayed in Table 2.

**Table 2:** Baseline characteristics of include studies and methodological assessment.

Authors	Study period	Country	Registration number	Study design	Type of IUD	Patients (n)	Timing of IUD insertion		Follow-up (months)
						Immediate/delayed	Immediate	Delayed	
Saav et al.^19^	2007.2–2010.10	Sweden	NCT01537562	RCT	Cu-IUD or LNG-IUS	62/54	5–9 days after medical abortion	21–35 days after medical abortion	6 months
Bednarek et al.^9^	2007.5–2008.12	USA	NCT00562276	RCT	LNG-IUS or Cu-IUD	258/317	Within 15 min after surgical abortion	2–6 weeks after surgical abortion	6 months
Constant et al.^20^	2018.8–2019.12	South Africa	NCT03505047	RCT	Cu-IUD	55/57	Within 24 h after medical abortion	3 weeks after medical abortion	6 months
Cremer et al.^21^	2007.4–2009.8	USA	#00540046	RCT	Cu-IUD	104/111	Within 15 min after surgical abortion	2–4 weeks after surgical abortion	6 months
Hogmark et al.^8^	2019.1–2021.2	Sweden	NCT03603145	RCT	Cu-IUD or LNG-IUS	117/118	Within 48 h after medical abortion	2–4 weeks after medical abortion	6 months
Hohmann et al.^22^	2007.2–2009.4	USA		RCT	LNG-IUS	44/44	Immediate after surgical abortion	3–6 weeks after surgical abortion	6 months
Korjamo et al.1^23^	2013.1–2014.12	Finland	NCT01755715	RCT	LNG-IUS	27/28	0–3 days after medical abortion	2–4 weeks after medical abortion	12 months
Korjamo et al. 2(a)^24^	2013.1–2014.12	Finland	NCT01755715	RCT	LNG-IUS	51/50	Immediate (same day) after medical abortion	2–4 weeks after medical abortion	12 months
Korjamo et al. 2(b)^24^	2013.1–2014.12	Finland	NCT01755715	RCT	LNG-IUS	27/28	Immediate (same day) after medical abortion	2–4 weeks after medical abortion	12 months
Korjamo et al.3^25^	2013.1–2013.5	Finland	NCT01755715	RCT	LNG IUS	55/53	Within 3 days after medical abortion	2–4 weeks after medical abortion	12 months
Shimoni et al.^26^	2008.7–2009.10	USA	NCT00737178	RCT	Cu-IUD	71/85	Within 1 week after medical abortion	4–6 weeks after medical abortion	6 months

*RCT* randomised controlled trial, *LNG-IUS* levonorgestrel intrauterine system, *Cu-IUD* copper intrauterine device, *MTOP* medical termination of pregnancy.

### Demographic overview

The two groups displayed no significant difference in terms of age (WMD: 0.30; 95% CI − 0.25, 0.85; *P* = 0.29), BMI (WMD: 0.10; 95% CI − 0.48, 0.68; *P* = 0.73), gestational age at medical abortion (WMD: 0.05; 95% CI − 1.32, 1.41; *P* = 0.95), past pregnancy (≥ 1) (RR: 1.02; 95% CI 0.97, 1.07; *P* = 0.53), parity (≥ 1) (RR: 1.03; 95% CI 0.97, 1.10; *P* = 0.32), and prior abortion (≥ 1) (RR: 1.00; 95% CI 0.90, 1.12; *P* = 0.97) (Table 3).

**Table 3:** Demographics and clinical characteristics of included studies.

Outcomes	Studies	No. of patients	WMD or RR	95% CI	p-value	Heterogeneity			
		Immediate/delayed				Chi^2^	df	p-value	*I*^2^ (%)
Age (years)	11	977/1048	0.30	[− 0.25,0.85]	0.29	6.12	10	0.81	0
BMI (kg/m^2^)	5	524/579	0.10	[− 0.48,0.68]	0.73	1.64	4	0.80	0
Gestational age at medical abortion(days)	8	572/578	0.05	[− 1.32,1.41]	0.95	14.71	7	0.04	52
Past pregnancy (≥ 1)	7	715/784	1.00	[0.95,1.06]	0.90	2.72	6	0.84	0
Parity (≥ 1)	10	860/930	1.03	[0.96,1.10]	0.40	5.24	9	0.81	0
Prior abortion (≥ 1)	8	742/800	1.00	[0.90,1.12]	0.99	4.29	7	0.75	0

*BMI* body mass index, *WMD* weighted mean difference, *RR* relative risk, *CI* confidence interval.

### IUD inserted

Three studies showed successful IUD insertions (IUD inserted after the expulsion of the conception product) in participants after surgical abortion and eight studies after medical abortion.

**Figure 4:**
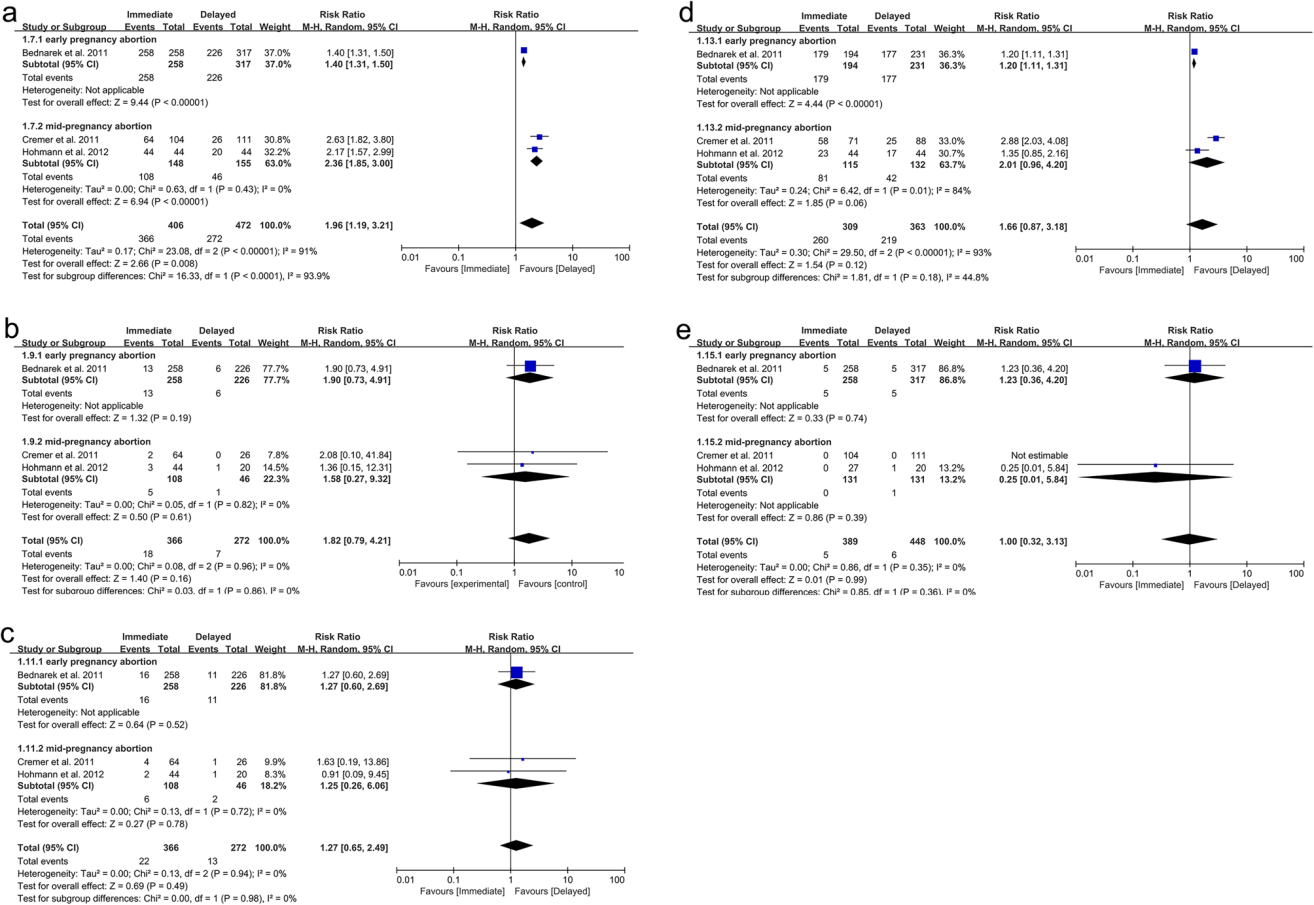
Forest plot based on surgical abortion. (**a**) IUD inserted. (**b**) IUD expelled at 6 months. (**c**) IUD removed at 6 months. (**d**) IUD used at 6 months (**e**) infection.

As for IUD insertion after surgical abortions, 878 patients were included (406 in the immediate placement group and 472 in the delayed placement group)^9,21,22^. The pooled analysis showed that the immediate placement group had a significantly higher rate of successful insertion than the delayed placement group (RR: 1.96; 95% CI 1.19, 3.21; *P* = 0.008), with significant heterogeneity (I^2^ = 91%, *P* < 0.00001) (Fig. 4a). Visualization assessment via funnel plot showed no significant publication bias (Fig. 6a). Egger's test was not statistically significant (*P* = 0.061). Subgroup analysis by gestational week at abortion revealed *P* < 0.00001 in both groups. To identify the source of heterogeneity, we conducted a sensitivity analysis. The results were unstable; after excluding the data from the study by Hohmann et al., the statistical results became insignificant (Fig. 1a). Additionally, we determined the source of heterogeneity. By excluding the article by Bednarek et al., the heterogeneity decreased from 91 to 0%, suggesting that this might be the source of the heterogeneity.

**Figure 5:**
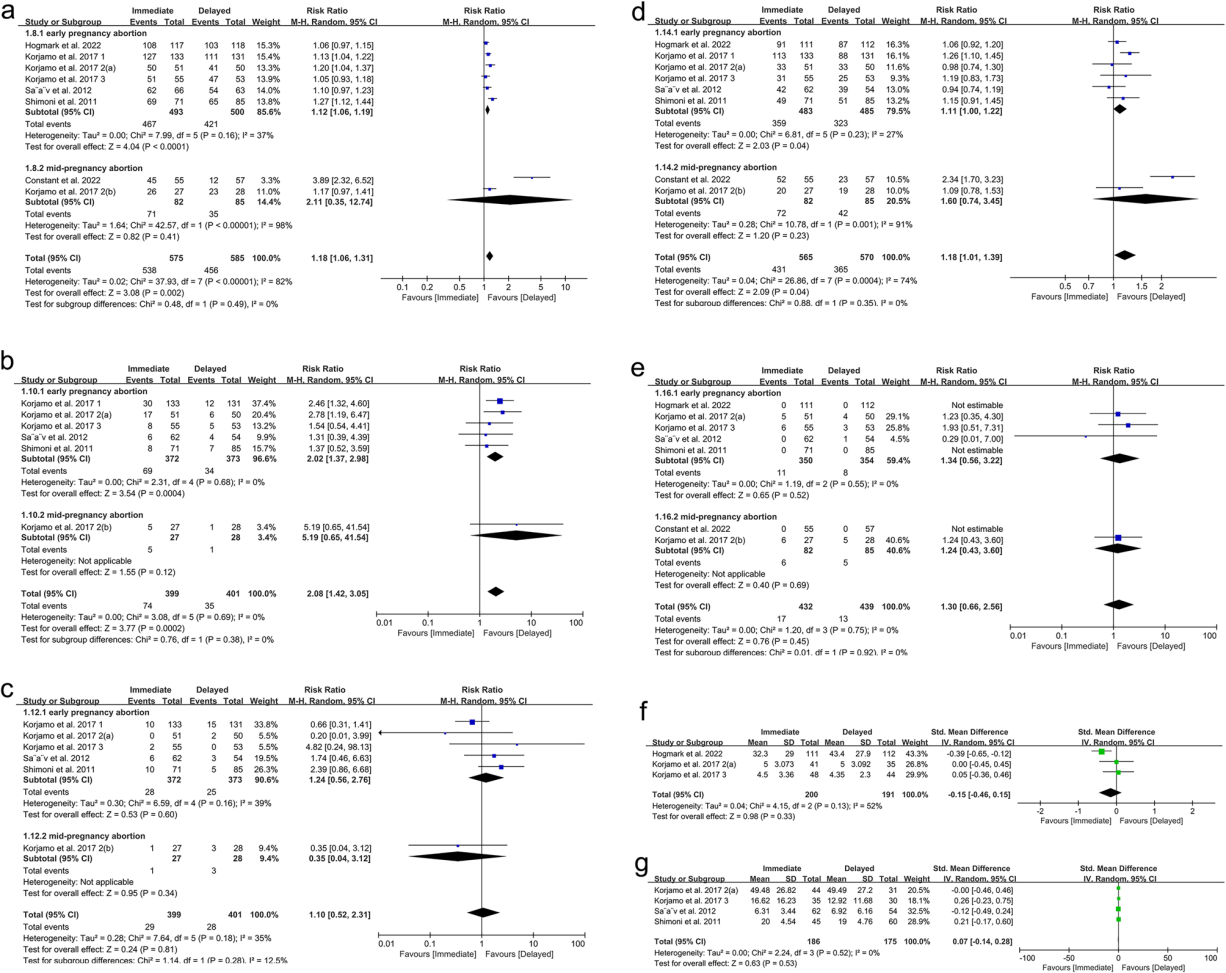
Forest plot based on medical abortion. (**a**) IUD inserted. (**b**) IUD expelled at 6 months or 1 year. (**c**) IUD removed at 6 months or 1 year. (**d**) IUD used at 6 months or 1 year. (**e**) Infection. (**f**) Pain score at IUD insertion (VAS or NRS). (**g**) Number of bleeding or spotting days during the reference period.

As for medical abortion, 1160 patients were included (575 in the immediate insertion group and 585 in the delayed insertion group)^8,19,20,23–26^. The combined analysis indicated a notably higher insertion rate in the immediate placement group than in the delayed group (RR: 1.18; 95% CI 1.06, 1.31; *P* = 0.002), with substantial heterogeneity (I^2^ = 82%, *P* < 0.00001) (Fig. 5a). The funnel plot showed publication bias^20^ (Fig. 7a). Egger's test showed statistical significance (*P* = 0.018), indicating publication bias. Subgroup analysis based on gestational weeks showed that the combined results for the early miscarriage group^8,19,23–26^ (RR: 1.12; 95% CI 1.06, 1.19; *P* < 0.0001) were statistically significant, with no apparent heterogeneity (I^2^ = 37%, *P* = 0.16). For the mid-term miscarriage group^20,24^ (RR: 2.11; 95% CI 0.35, 12.74; *P* = 0.41), there was no statistical difference, with significant heterogeneity (I^2^ = 98%, *P* < 0.00001). Subgroup analysis based on IUD type and region showed that the two studies^20,26^ using copper IUDs alone had no statistically significant differences (RR: 2.19; 95% CI 0.49, 9.67; *P* = 0.30). In the six studies conducted in Europe^8,19,23–25^, the heterogeneity of the combined results was 0%. The sensitivity analysis found the results were stable. After excluding the data from the article by Constant et al.^20^, the heterogeneity decreased from 82 to 27%, which may be the source of heterogeneity (Fig. 1b).

**Figure 6:**
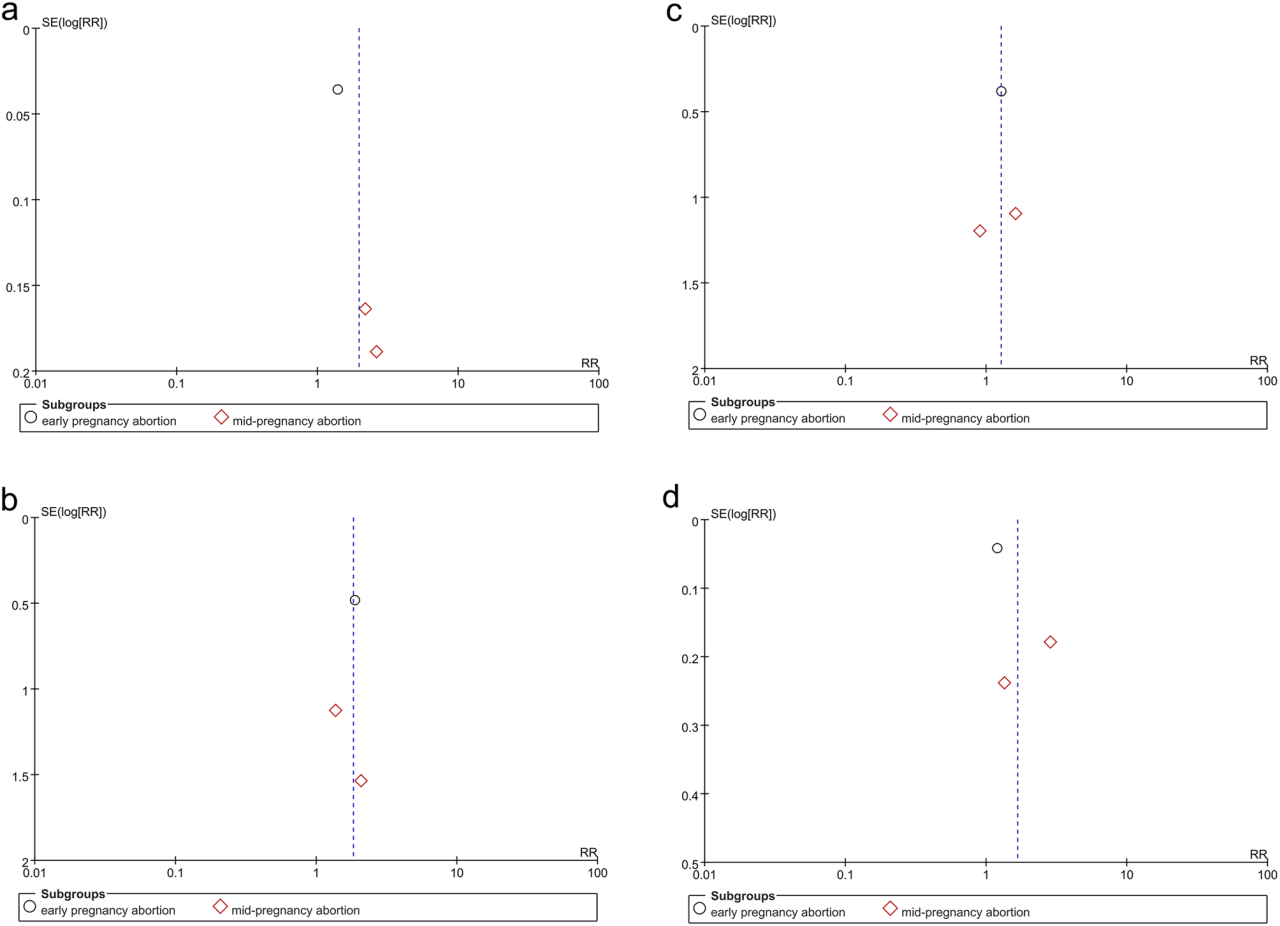
Funnel plot based on surgical abortion. (**a**) IUD inserted. (**b**) IUD expelled at 6 months. (**c**) IUD removed at 6 months. (**d**) IUD used at 6 months.

**Figure 7:**
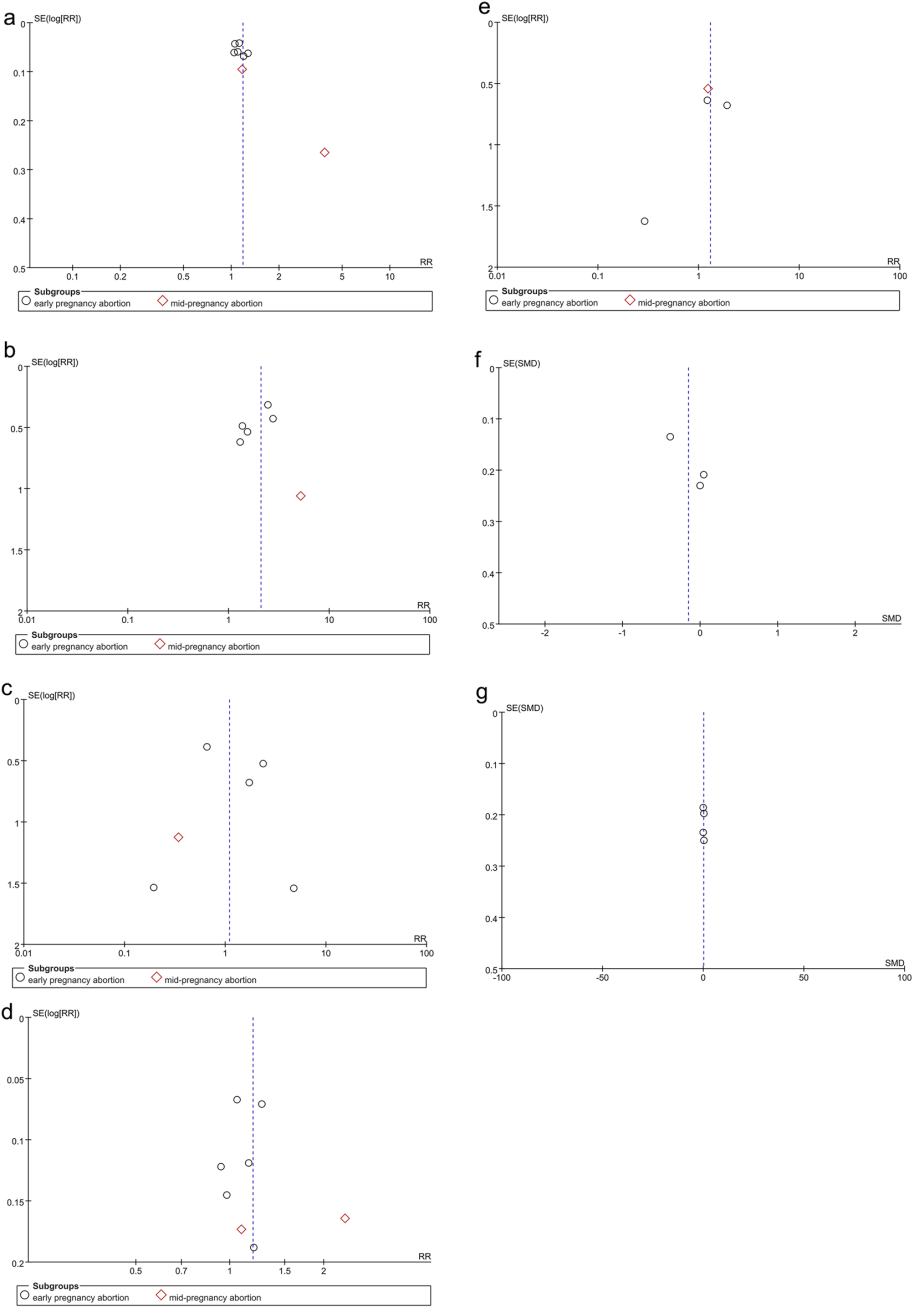
Funnel plot based on medical abortion. (**a**) IUD inserted. (**b**) IUD expelled at 6 months or 1 year. (**c**) IUD removed at 6 months or 1 year. (**d**) IUD used at 6 months or 1 year. (**e**) Infection. (**f**) Pain score at IUD insertion (VAS or NRS). (**g**) Number of bleeding or spotting days during the reference period.

### IUD expelled

Three studies analyzed the 6-month expulsion rate of IUDs in 638 subjects who underwent surgical abortion (366 in the immediate placement group and 272 in the delayed placement group)^9,21,22^. The meta-analysis showed no difference in expulsion rates between the immediate and delayed placement groups (RR: 1.82; 95% CI 0.79, 4.21; *P* = 0.16), with no significant heterogeneity (I^2^ = 0%, *P* = 0.96) (Fig. 4b). The funnel plot in Fig. 6b exhibits no discernible evidence of publication bias, and the results from Egger's test do not indicate a significant presence of publication bias (*P* = 0.744). Following subgrouping based on gestational weeks at abortion, the results also showed no significant statistical difference.

Six studies analyzed the expulsion rate of IUDs in 800 patients after medical abortion at 6 months or 1 year follow-up^19,23–26^. Among them, five studies reported the expulsion rate of IUDs after medical abortion in early pregnancy (372 in the immediate placement group and 373 in the delayed placement group)^19,23–26^. One study reported the expulsion rate of IUDs after medical abortion in mid-pregnancy (27 in the immediate placement group and 28 in the delayed placement group)^24^. The combined analysis showed that the expulsion rate in the immediate placement group was higher than that in the delayed placement group (RR: 2.08; 95% CI 1.42, 3.05; *P* = 0.0002), with no heterogeneity (I^2^ = 0%, *P* = 0.69) (Fig. 5b). The funnel plot showed no apparent publication bias (Fig. 7b), and Egger's test did not reveal any substantial publication bias (*P* = 0.870). In the subgroup analysis by IUD type and region, one study conducted in the USA using only Cu-IUDs showed no statistically significant difference^26^ (RR: 1.37; 95% CI 0.52, 3.59; *P* = 0.52). Similarly, one study using LNG-IUS or Cu-IUD observed no statistical significance^19^ (RR: 1.31; 95% CI 0.39, 4.39; *P* = 0.67) (Table 1).

### IUD removed

In the three studies^9,21,22^ on IUD removal rates at 6 months after surgical abortion, no significant difference was noted in the combined results (RR: 1.27; 95% CI 0.65, 2.49; *P* = 0.49) (Fig. 4c). The funnel plot in Fig. 6c did not show any significant publication bias, and Egger's test also found no publication bias (*P* = 0.911).

In six follow-up studies^19,23–26^ on the removal rates of IUDs 6 months or 1 year after medical abortions, five studies were for early pregnancy abortion groups (372 cases in the immediate placement group and 373 cases in the delayed placement group)^19,23–26^, and one study was for mid-pregnancy abortion groups (27 cases in the immediate placement group and 28 cases in the delayed placement group)^24^. The combined results showed no marked difference in the IUD removal rates between the two groups (RR: 1.10; 95% CI 0.52, 2.31; *P* = 0.81), with no significant heterogeneity (I^2^ = 35%, *P* = 0.18) (Fig. 5c). The funnel plot showed no obvious publication bias (Fig. 7c). Egger's test revealed no significant publication bias (*P* = 0.996).

### IUD used

Three studies reported the number of IUD users at 6 months after surgical abortion, including 672 women (309 in the immediate group and 363 in the delayed group)^9,21,22^. The difference between the two groups was not statistically significant (RR: 1.66; 95% CI 0.87, 3.18; *P* = 0.12), with significant heterogeneity (I^2^ = 93%, *P* < 0.00001) (Fig. 4d). The funnel plot showed no obvious publication bias (Fig. 6d). Egger's test did not find significant publication bias (*P* = 0.477). Our sensitivity analysis results were unstable. After excluding the article by Cremer et al.^21^, the heterogeneity decreased from 93 to 0%, indicating this article may be the source of the heterogeneity (Fig. 1c).

Eight studies reported the number of IUD users at 6 months or 1 year after medical abortion, involving 1135 patients (565 in the immediate group and 570 in the delayed group)^8,19,20,23–26^. The difference between the two groups was statistically significant (RR: 1.18; 95% CI 1.01, 1.39; *P* = 0.04). The immediate placement group had a higher usage rate at 6 months or 1 year than the delayed placement group, with considerable heterogeneity (I^2^ = 74%, *P* = 0.0004) (Fig. 5d). The funnel plot showed no obvious publication bias (Fig. 7d). Egger's test did not find significant publication bias (*P* = 0.627). Subgroup analysis based on gestational weeks at abortion showed that, in the early pregnancy abortion group, the number of IUD users at 6 months or 1 year after medical abortion was higher in the immediate group than that in the delayed group, with significant differences (RR: 1.11; 95% CI 1.00, 1.22; *P* = 0.04) and insignificant heterogeneity (I^2^ = 27%, *P* = 0.23) (Fig. 5d). In the mid-pregnancy abortion group, there was no statistically significant difference between the two groups (RR: 1.60; 95% CI 0.74, 3.45; *P* = 0.23), with significant heterogeneity (I^2^ = 91%, *P* = 0.001).

Subgroup analyses based on IUD type and region displayed no statistically significant differences in the two studies^20,26^ that exclusively used Cu-IUD (RR: 1.63; 95% CI 0.80, 3.29; *P* = 0.18). Similarly, there was no marked difference in the two studies^8,19^ using either LNG-IUS or Cu-IUD (RR: 1.03; 95% CI 0.91, 1.15; *P* = 0.66). In two studies^20,26^ conducted in Africa and the United States, as well as six combined studies^8,19,23–25^ in Europe, there were also no statistically significant differences in outcomes (Table 1). The sensitivity analysis revealed unstable results. After excluding the study by Constant et al.^20^, heterogeneity decreased to 12%, indicating that this study was a potential source of heterogeneity (Fig. 1d).

### Infection

The combined results for infections after immediate versus delayed IUD insertion following surgical abortion showed no significant statistical difference (RR: 1.00; 95% CI 0.32, 3.13; *P* = 0.99) (Fig. 4e).

Following medical abortion, the combined results for infections after IUD insertion also showed no statistical difference (RR: 1.30; 95% CI 0.66, 2.56; *P* = 0.45). There was no statistical difference in the outcomes between the early pregnancy abortion group and the mid-pregnancy abortion group (Fig. 5e). The funnel plot showed visual publication bias (Fig. 7e). However, Egger's test found no statistical significance (P = 0.265), suggesting no apparent publication bias.

### Pain score at IUD insertion (VAS or NRS) after medical abortion

In studies on pain scores after IUD insertion following medical abortion, 3 studies were included, with 391 patients (200 in the immediate group and 191 in the delayed group)^8,24,25^. The pooled evidence revealed no statistically significant difference between the immediate and delayed IUD placement groups (SMD − 0.15; 95% CI − 0.46, 0.15; *P* = 0.33), affirming the absence of significant differences in IUD insertion pain. However, notable heterogeneity was observed (I^2^ = 52%, *P* = 0.13) (Fig. 5f). The funnel plot in Fig. 7f and Egger's test (*P* = 0.159) did not show significant publication bias. In subgroup analyses based on IUD type and region, one study^8^ using LNG-IUS or Cu-IUD showed significant differences in pain scores (SMD: − 0.39; 95% CI − 0.65, − 0.12; *P* = 0.004) (Table 1). Our sensitivity analysis showed robust results. After excluding the article by Hogmark et al.^8^, heterogeneity decreased from 52 to 0%, suggesting that this study might be the source of the heterogeneity (Fig. 1e).

### Number of bleeding or spotting days during the reference period after medical abortion

Data on bleeding or spotting within the reference period were obtained from four studies, including 361 patients (186 in the immediate group and 175 in the delayed group)^19,24–26^. The two groups exhibited no statistically significant difference (SMD: 0.07; 95% CI − 0.14, 0.28; *P* = 0.53), nor any substantial heterogeneity (I^2^ = 0%, *P* = 0.52) (Fig. 5g). The funnel plot showed no significant publication bias (Fig. 7g).

### Uterine perforation

No severe adverse events like uterine perforation were observed in any of the included studies.

## Discussion

IUDs are regarded as highly effective contraceptive devices when correctly inserted and used. They offer excellent protection against pregnancy with minimal systemic side effects^27,28^. Their effectiveness, safety, and satisfaction have been confirmed across various populations, including multiparous, nulliparous, and young women^29^. In the past few years, there has been ongoing research on the advantages and disadvantages of immediate post-abortion IUD placement versus delayed placement. This topic has generated controversy worldwide^10,30^. Therefore, we conducted this systematic review and meta-analysis, which revealed several important findings.

Since the abortion method may significantly impact the outcomes of IUD placement, we analyzed the data separately for surgical abortions and medical abortions and made comparative analyses based on the gestational week at abortion. For surgical abortions, our combined data showed significantly higher IUD insertion rates in the immediate placement group than that in the delayed placement group. However, no statistically significant differences were noticed in IUD expulsion rate, removal rate, IUD usage rates after 6 months, or infection rate post-insertion, which may be related to the limited included studies. Previous studies have indicated that immediate IUD insertion following surgical abortion is safe and effective^31,32^, particularly in IUD insertion rates and usage rates, despite reports of higher expulsion rates. This method is still worth promoting^32^. Concerning medical abortion, we first found that the immediate placement group had a higher IUD insertion rate, especially in the early pregnancy abortion group. Meanwhile, the expulsion rates of IUDs after 6 months or 1 year after medical abortion were higher in the immediate placement group than in the delayed group, but the usage rates after 6 months or 1 year were relatively higher in the immediate group. Our results also showed no significant differences between the two groups in removal rates, infection rates, pain scores during insertion, and bleeding days post-insertion after 6 months or 1 year of medical abortion IUD placement.

Sensitivity and subgroup analyses were performed on results with heterogeneity to identify potential sources of heterogeneity. As for surgical abortion, due to the limited included studies, the results might be related to a higher rate of follow-up loss^20,21^. In the sensitivity analysis of medical abortions, the outcomes of post-abortion IUD placement were relatively robust, and the heterogeneity might be related to the influence of mid-trimester abortions and the potential impact of contraceptive device type (Cu-IUD) as suggested by Constant et al.^20^. For the heterogeneity in pain scores after IUD insertion following medical abortions, the results of the sensitivity analysis were relatively robust. We also identified that heterogeneity may stem from the study of Hogmark et al.^8^, which may be related to inconsistent pain scoring criteria and significant individual differences in pain scores. More studies are required for further analysis.

Here are discussions about the related mechanisms: 1. IUD insertion immediately after an abortion ensures prompt contraception and reduces the risk of subsequent pregnancies^33^. This approach also avoids the need for follow-up visits, which are a common obstacle to post-treatment care. Immediate IUD placement offers several benefits, such as minimizing patient visits and healthcare costs, thereby enhancing its feasibility^34^. 2. Surgical abortion requires the removal of uterine contents, and because the cervix dilates during surgery, it provides a unique opportunity for immediate IUD placement. However, this method increased the risk of uterine perforation, particularly post mid-trimester abortion. Additionally, abortions at the early gestational stage may lead to increased IUD expulsion rates followed by immediate insertion. Medical abortion, which is accomplished through the use of pharmaceuticals, induces uterine contractions and cervical dilation, potentially leading to an increase in IUD expulsion rates^35^. After an abortion, the uterus usually needs a long recovery period before IUD placement, a process that can be further complicated following a mid-trimester abortion. In such cases, inadequate uterine contraction or incomplete cervical dilation may prevent immediate IUD insertion. Gestational age also matters: the physiological consequences of abortions in the early (pre-12 weeks) and mid-trimester (post-12 weeks) stages are significantly different. After an early abortion, the smaller uterus and rapid cervical closure present obstacles for IUD insertion, particularly after medical abortions. In contrast, mid-trimester abortions, characterized by an enlarged uterine size and extensive cervical dilation, may simplify IUD placement but also increase the risk of uterine perforation. 3. Copper IUDs (Cu-IUDs) and hormone-releasing IUDs (LNG-IUDs) are the predominant types of IUDs^36^. Cu-IUDs exert their contraceptive effects by inducing a local inflammatory response that is detrimental to sperm and ova. However, the post-abortion uterine environment might impede IUD retention, raising the risk of expulsion^37^. In contrast, LNG-IUDs, due to their size and the effect of levonorgestrel on the endometrium, may be more suitable for immediate placement^38,39^. 4. Our subgroup analysis indicated regional differences in IUD use, with immediate placement being more prevalent in Europe and North America^40^, possibly influenced by healthcare policies and accessibility. These differences underscore the need for region-specific strategies in post-abortion contraceptive care, taking into account local healthcare systems and cultural contexts. 5. Concerning infection risks, our aggregated data suggested that immediate post-abortion IUD placement did not markedly elevate infection risks. However, it is important to consider the specific clinical backgrounds when making decisions.

Consistent with previous findings, our results showed significant advantages of immediate IUD placement post-abortion. Bednarek et al.^9^ indicated that women who received IUD insertion immediately after an abortion had higher insertion rates and higher usage rates at 6 months. Conversely, Hohmann et al.^22^ did not observe statistically significant differences in IUD usage rates at 6 months, potentially attributed to a substantial loss to follow-up that hinders the ability to identify differences in IUD usage rates. Therefore, meticulous tracking and follow-up are of considerable importance in research.

Our study has some limitations. First, our meta-analysis only included 11 studies, with just three on surgical abortion and eight on medical abortion, which might be relatively small. Additionally, significant heterogeneity was found in five outcomes. Although sensitivity analyses were performed to assess the robustness of the results and identified some potential sources of heterogeneity, some sources remain uncertain. Given the potential confounding factors, the results of this meta-analysis should be interpreted with caution. Finally, due to the lack of data on subsequent pregnancies and satisfaction with IUDs, we were unable to comprehensively assess the outcomes of IUDs in both groups. In the future, more extensive and well-planned RCTs and long-term follow-ups are needed to further compare successful insertion rates, expulsion rates, removal rates, usage rates during follow-up, satisfaction, and adverse reactions between immediate and delayed IUD insertion after abortions. Consideration could also be given to adopting more advantageous surgical techniques, such as the rational use of hysteroscopy, to improve IUD insertion^41,42^.

## Conclusion

Compared to delayed placement, immediate insertion of IUDs can not only increase the usage rate at 6 months or 1 year but also enhance the placement rate. Nonetheless, clinicians must modify the insertion approach based on their expertise and the patient's particular circumstances.

### Supplementary Information

Supplementary Information 1.

Supplementary Information 2.

## Data Availability

The original data supporting the conclusions of this article will be provided by the authors without any reservations. Xuejing Jin should be contacted if someone wants to request the data from this study.
