# Supplementary material for: Immediate and delayed placement of the intrauterine device after abortion: a systematic review and meta-analysis
Source: Sci Rep. 2024 May 18;14:11385. doi: 10.1038/s41598-024-62327-1 (PMC11102502; doi:10.1038/s41598-024-62327-1)
Supplement: Supplementary file 2 — Supplementary Information 2. [file 41598_2024_62327_MOESM2_ESM.docx]

| **Online Resource 2** Detailed search strategy in three databases | | |
| --- | --- | --- |
| Database | No | Search strategy |
| Pubmed | 1 | ("Intrauterine Devices"[Mesh]) OR ((((((((((((((((Device, Intrauterine[Title/Abstract]) OR (Devices, Intrauterine[Title/Abstract])) OR (Intrauterine Device[Title/Abstract])) OR (Contraceptive IUDs[Title/Abstract])) OR (Contraceptive IUD[Title/Abstract])) OR (IUD, Contraceptive[Title/Abstract])) OR (IUDs, Contraceptive[Title/Abstract])) OR (Contraceptive Devices, Intrauterine[Title/Abstract])) OR (Contraceptive Device, Intrauterine[Title/Abstract])) OR (Device, Intrauterine Contraceptive[Title/Abstract])) OR (Devices, Intrauterine Contraceptive[Title/Abstract])) OR (Intrauterine Contraceptive Device[Title/Abstract])) OR (Intrauterine Contraceptive Devices[Title/Abstract])) OR (Unmedicated IUDs[Title/Abstract])) OR (Unmedicated IUD[Title/Abstract])) OR (IUD, Unmedicated[Title/Abstract])) |
|  | 2 | abortion |
|  | 3 | 1 and 2 |
| Embase* | 1 | Intrauterine Devices.af. or Device, Intrauterine.ab. or Devices, Intrauterine.ab. or Intrauterine Device.ab. or Contraceptive IUDs.ab. or Contraceptive IUD.ab. or IUD, Contraceptive.ab. or IUDs, Contraceptive.ab. or Contraceptive Devices, Intrauterine.ab. or Contraceptive Device, Intrauterine.ab. or Device, Intrauterine Contraceptive.ab. or Devices, Intrauterine Contraceptive.ab. or Intrauterine Contraceptive Device.ab. or Intrauterine Contraceptive Devices.ab. or Unmedicated IUDs.ab. or Unmedicated IUD.ab. or IUD, Unmedicatedc.ab. |
|  | 2 | abortion.af. |
|  | 3 | 1 and 2 |
| Cochrane | 1 | Intrauterine Devices.af. or Device, Intrauterine.ab. or Devices, Intrauterine.ab. or Intrauterine Device.ab. or Contraceptive IUDs.ab. or Contraceptive IUD.ab. or IUD, Contraceptive.ab. or IUDs, Contraceptive.ab. or Contraceptive Devices, Intrauterine.ab. or Contraceptive Device, Intrauterine.ab. or Device, Intrauterine Contraceptive.ab. or Devices, Intrauterine Contraceptive.ab. or Intrauterine Contraceptive Device.ab. or Intrauterine Contraceptive Devices.ab. or Unmedicated IUDs.ab. or Unmedicated IUD.ab. or IUD, Unmedicatedc.ab. |
|  | 2 | abortion.af. |
|  | 3 | 1 and 2 |
| Web of science | 1 | Intrauterine Devices (All Fields) or Device, Intrauterine (Abstract) or Devices, Intrauterine (Abstract) or Intrauterine Device (Abstract) or Contraceptive IUDs (Abstract) or Contraceptive IUD (Abstract) or IUD, Contraceptive (Abstract) or IUDs, Contraceptive (Abstract) or Contraceptive Devices, Intrauterine (Abstract) or Contraceptive Device, Intrauterine (Abstract) or Device, Intrauterine Contraceptive (Abstract) or Devices, Intrauterine Contraceptive (Abstract) or Intrauterine Contraceptive Device (Abstract) or Intrauterine Contraceptive Devices (Abstract) or Unmedicated IUDs (Abstract) or Unmedicated IUD (Abstract) or IUD, Unmedicated (Abstract) |
|  | 2 | abortion (All Fields) |
|  | 3 | 1 and 2 |
| CNKI | 1 | Intrauterine device + Intrauterine ring + IUDs + Contraceptive device + Contraceptive device placement surgery |
|  | 2 | Miscarriage + Abortion + Surgical abortion + Medical abortion |
|  | 3 | 1 and 2 |
| Wanfang database | 1 | Intrauterine device OR Intrauterine ring OR IUDs OR Contraceptive device OR Contraceptive device placement surgery |
|  | 2 | Miscarriage OR Abortion OR Surgical abortion OR Medical abortion |
|  | 3 | 1 and 2 |
| * We retrieved articles from Embase via the Ovid (https://ovidsp.ovid.com/). | | |
